# Study on the Quality, Relevance, and Comparability of YouTube Videos Expressing Stroke and Its Consequences From Various Sources

**DOI:** 10.7759/cureus.43277

**Published:** 2023-08-10

**Authors:** Ankita Nanda, Manoj M N, Geethiikha Jammula, Udvas Sen, Nikhitha Konda, Iorwuese Ali Daniel, Rachel Mary Manoj

**Affiliations:** 1 Internal Medicine, Ranga Raya Medical College, Kakinada, IND; 2 Internal Medicine, Bangalore Medical College and Research Institute, Bangalore, IND; 3 Internal Medicine, Guntur Medical College, Guntur, IND; 4 Internal Medicine, Agartala Government Medical College, Agartala, IND; 5 Internal Medicine, Alluri Sitaramaraju Academy of Medical Sciences, Eluru, IND; 6 Internal Medicine, Sohar Extended Health Center, Sohar, OMN; 7 Internal Medicine, Nicolae Testemițanu State University of Medicine and Pharmacy, Chisinău, MDA

**Keywords:** stroke cause, stroke support, stroke cure, stroke prevention, stroke treatment, paralysis, stroke

## Abstract

Background and objective

Stroke-related deaths have been one of the major causes of death worldwide due to its rising risk factors. As a result, several people rely on YouTube as a great source of information on stroke without knowing the genuineness of the content. This study aims to assess the quality and reliability of the information on stroke uploaded on the YouTube platform using the Global Quality score (GQS) and DISCERN score (DS), respectively.

Methodology

A cross-sectional observational study was conducted in April 2023. Stroke-related keywords were used to search for videos on YouTube. Videos that met inclusion criteria were evaluated for baseline characteristics (likes, comments, views, duration of video, time since posted, and uploader type) and type of information in the video about stroke (symptoms, etiology, treatment, and other parameters). These videos were then evaluated for quality and reliability of information using GQS and DS, respectively.

Results

After applying inclusion and exclusion criteria and removing the duplicates, 73 YouTube videos were selected. The videos had a total number of 23,927,445 views, 385,324 likes, and 31,927 comments. Maximum videos were uploaded by hospitals (25, 34.2%). Several videos described the symptoms (54, 73.97%), treatment (50, 68.49%), and etiology (49, 67.12%) of stroke. The reach of videos measured by the Video Power Index (VPI) was highest for videos uploaded by healthcare organizations (mean VPI = 120.11). There was no statistically significant difference (*P* > 0.05) in the quality (GQS score) and reliability (DS) of videos uploaded by doctors, hospitals, healthcare organizations, and news channels.

Conclusions

YouTube can become an important source to disseminate information about health-related conditions like stroke if the videos uploaded are of high quality (GQS score) and reliable (DS).

## Introduction

The World Health Organization has defined a stroke as a rapidly developing clinical sign of focal or global disturbance of cerebral function, lasting more than 24 hours or leading to death, with no apparent cause other than vascular origin [1]. As per the World Stroke Organization - Global Stroke Fact Sheet 2022, stroke is the second-leading cause of death and the third-leading cause of death and disability combined in the world [2]. Nonmodifiable risk factors like age, ethnicity, genetics, and family history contribute to stroke risk [3], but there are disparities in stroke risk, rates, and treatment worldwide.

YouTube, the second-most-popular video-sharing platform in the world, with over two billion subscribers, has become a popular platform to disseminate health information [4-6]. Social media platforms like YouTube have been evaluated for validity and dependability for several diseases like glioblastoma, abdominal aortic aneurysms, and chronic obstructive pulmonary disease [7-12]. Stroke, the fifth-leading cause of mortality in the United States, is still not being given enough consideration in this regard [13]. The lack of reliable information on stroke on platforms like YouTube highlights the need for video analysis and implementation of an audience engagement criterion for video creators to guarantee that viewers get accurate education, particularly since stroke is a medical emergency requiring immediate care to minimize the damage [14]. Therefore, this study aims to assess the reliability and quality of stroke information presented on YouTube.

This study aims to assess the reliability and quality of the information on stroke on YouTube using the DISCERN score (DS) and the Global Quality score (GQS), respectively.

## Materials and methods

This research was a cross-sectional observational study carried out in April 2023. Our study did not require institutional permission because there were no human participants.

A Google Forms survey was designed with preset parameters, concentrating on three main traits. As a starting point, we looked into the fundamental details of the videos, such as the origin of the uploaded videos and the posting period. Second, we looked for descriptions of symptoms, causes, investigations, prevention, therapy, mortality, rehabilitation, and personal stories from patients and their families as we examined the informational content of the videos. We also took into account whether the article included material that was sponsored by medical professionals or pharmaceutical firms. Following this, the quality and reliability of each video were evaluated using GQS [15] and DS [16]. Using keywords such as stroke, paralysis, stroke treatment, stroke prevention, stroke cause, stroke cure, and stroke support, each of the seven authors searched for and analyzed 15 YouTube videos (Appendix).

The YouTube videos that specifically addressed symptoms, causes, and treatment options of stroke; had a duration of at least two minutes (too short to convey information) but did not exceed 20 minutes (does not hold the viewer's attention); and were in English language (to maintain a homogeneous set of videos) were included in this study. YouTube videos that were unrelated to stroke, shorter than two minutes or longer than 20 minutes, and in language other than English were excluded from the study. Any duplicate entries were excluded from the study.

The information gathered from the selected videos was entered into Google Sheets and later transferred to Microsoft Excel for further analysis (Appendix). Statistical analysis was conducted using IBM SPSS Statistics for Windows (Version 21.0, IBM Corp., Armonk, NY, USA).

## Results

For this study, a total of 105 videos (15 videos by each of the seven authors) were evaluated. However, after applying the inclusion and exclusion criteria and removing duplicate videos, only 73 videos were included in this study. The popularity of the videos was assessed based on several characteristics, such as the number of views, likes, dislikes, and comments.

Table 1 highlights the popularity of the videos evaluated. The total number of views, likes, and comments were 23,927,445, 385,324, and 31,927, respectively.

**Table 1:** Popularity of the videos evaluated.

Characteristic of the videos	Number
Total no. of views	23,927,445
Total no. of likes	385,324
Total no. of dislikes	33,542
Total no. of comments	31,927

Table 2 highlights the duration since the video was uploaded and the source of information. The majority (62, 84.9%) of the videos were uploaded more than a year ago, with 40 (54.7%) being uploaded by doctors and healthcare organizations combined, outnumbering those uploaded by news channels by 10 (13.7%) and others by 20 (27.4%).

**Table 2:** Characteristics of the YouTube videos analyzed.

Characteristics of YouTube videos	*n* (%)
Time since upload	
More than a month to six months (31-180 days)	04 (5.5)
More than six months to last one year (180-365 days)	07 (9.6)
More than one year (>365 days)	62 (84.9)
Type of uploader	
Doctor	15 (20.5)
Hospital	25 (34.2)
Healthcare organization	03 (4.1)
News channel	10 (13.7)
Other	20 (27.4)

Table 3 highlights how most of the videos discussed the etiology and treatment of the disease. A single video can discuss multiple types of information as well. A total of 54 (73.97%) videos discussed symptoms, 50 (68.49%) about treatment, and 49 (67.12%) about cause or etiology. Only 20 (27.4%) videos discussed mortality, while 19 (26.03%) videos had information about rehabilitation, 16 (21.92%) videos had patient sharing their own experience, 9 (12.33%) videos had parents sharing their experience with their family members, 12 (16.44%) videos had promotional content, and 4 (5.48%) videos had information about support groups.

**Table 3:** Information about strokes in the YouTube videos.

Type of information provided by the YouTube videos	*n* (%)
Description of symptoms	54 (73.97)
Information about cause/etiology	49 (67.12)
Information about investigations/tests	32 (46.58)
Information about prevention/vaccines	34 (46.58)
Information about treatment	50 (68.49)
Information about mortality	20 (27.4)
Information about rehabilitation	19 (26.03)
Information about support groups	04 (05.48)
Information about people/patients sharing their own experience	16 (21.92)
Information about parents sharing their experience with their family members	09 (12.33)
Does the post have promotional content by pharmaceutical companies or by doctors?	12 (16.44)

Table 4 compares the quality and reliability of stroke information on YouTube between various groups of uploaders. The median Video Power Index (VPI) of videos uploaded by healthcare organization (median VPI = 120.11) and doctors (median VPI = 81.62) were higher compared to the other groups, suggesting a higher reach to the general population. However, this difference was not statistically significant (*P *> 0.05).

**Table 4:** Comparison of GQS, DS, and VPI based on the type of uploader. a and b signify that the values are too small and hence insignificant and DS is reliability score. *P* < 0.05 is significant. VPI, Video Popularity Index; GQS, Global Quality score; DS, DISCERN score

Assessment tools	Doctors (*n *= 15)	Hospitals (*n *= 25)	Healthcare organization (*n *= 3)	News agency (*n *= 10)	Others (*n *= 20)	*P*-value
	Median (IQ1, IQ3)	Median (IQ1, IQ3)	Median (IQ1, IQ3)	Median (IQ1, IQ3)	Median (IQ1, IQ3)	Kruskal-Wallis test
VPI	81.62 (11.56, 1872.94)	32.35 (12.07, 56.17)	120.11 (15.24, a)	39.74 (7.68, 73.42)	53.82 (6.08, 113.58)	0.326
GQS	4 (4, 5)	4 (3, 5)	4 (3, b)	4 (3, 4.25)	4 (3, 4.75)	0.727
DS	4 (3, 4)	4 (3, 4.5)	4 (4, 4)	4 (2, 5)	3 (3, 4)	0.864

The median GQS score was 4 for all the groups, which signifies that there was no significant difference in the quality of videos uploaded by the groups (Table 4).

The median DS was higher for doctors, hospitals, and healthcare organization (median DS = 4), suggesting a higher reliability of such videos compared to other groups. But the difference was not statistically significant (*P *> 0.05; Table 4).

## Discussion

YouTube has emerged as a highly popular platform for accessing medical information in recent times. Before seeking professional help, numerous patients rely on the Internet as a valuable and reliable source of health-related information [17]. As the second most widely utilized search engine on the internet, YouTube plays a significant role in facilitating the acquisition of such information. This content has the potential to assist individuals in enhancing their health management and stroke recovery [1]. In our study, the median quality score and reliability score of videos posted by doctors, hospitals, and healthcare organizations were higher compared to the other groups. However, it is important to recognize that YouTube still has certain limitations despite being a valuable resource for patients seeking information about stroke [13].

In this study, a total of 105 videos were evaluated; only 73 met the inclusion criteria, while in other studies, it was found that 101 out of 150 videos [13] and 21 out of 200 videos met the inclusion criteria [2].

In a study by Yasin and Altunisik in October 2019, it was found that the mean overall quality of YouTube videos according to the DS was of fair quality [17]. Whereas in this study, the median reliability score of videos uploaded by doctors, hospitals, and healthcare organizations was of good quality (DS = 4). In another study where 150 videos were analyzed, the reliability and quality analysis of YouTube was done using the DS and the *Journal of American Medical Association* (JAMA) score, respectively [17]. In this study, 105 videos were analyzed using GQS, DS, and VPI to compare and analyze the quality, reliability, and reach, respectively, of YouTube videos uploaded by different uploaders. It was found that a *P*-value > 0.05 indicates that there is no significant difference in the VPI, GQS, or reliability score among all the uploaders. The GQS of all types of uploaders was similar. Thus, in this study, it was reflected that overall, the quality of the content uploaded by different uploaders is of moderate to good quality.

In this study, the total number of views, likes, and comments were 23,927,445, 385,324, and 31,927, respectively, while in a study conducted by Yasin and Altunisik, the total number of views, likes, and comments were 115,875, 482, and 28, respectively [17].

However, every effort was made to include new videos, but only 11 (15.1%) were recently uploaded within a year. In this study, it was found that the majority of the videos (25, 34.2%) were uploaded by hospitals and 15 (20.5%) by doctors, compared to another study by Szmuda et al. where 66 (65.3%) videos were uploaded by hospitals and 31 (30.69%) were uploaded by educational channels [13]. This indicates that the videos uploaded by uploaders, such as news agencies and healthcare organizations, were overlooked by doctors and healthcare professionals, so these videos too are reliable and provide authentic information to the viewers. This also reflects the fact that many hospitals and educational YouTube channels were eager to educate their followers.

This study also showed that the most popular videos were those from healthcare organisations (high VPI). This was primarily because these channels or uploaders used animation and three-dimensional (3D) illustrations of the causes and mechanisms of stroke that were understandable to a common person. Furthermore, these channels placed a greater focus on developing intriguing content and applying high-quality animation to provide viewers with a pleasing audiovisual experience. However, videos from physicians or news channels were less popular because the information was conveyed verbally without any animation. Thus, scientists and news channels can reflect on this and apply visual techniques to attract more viewers. The ability of the internet and social media to connect and educate individuals about stroke provides possibilities to improve their quality of life and learn about novel treatments. The internet's easier access to information has also contributed to the globalization of medical knowledge and led to the development of new social support groups. Patients, particularly those who have impairments such as stroke, may discover information on novel treatments for neurological diseases online. Patients may even frequently turn to social media to manage their illnesses.

In this study, 54 (73.97%) videos discussed symptoms, 50 (68.49%) about treatment, and 49 (67.12%) about cause or etiology. Only 20 (27.4%) videos discussed mortality, while 19 (26.03%) videos had information about rehabilitation, 16 (21.92%) videos had patient sharing their own experience, 9 (12.33%) videos had parents sharing their experience with their family members, 12 (16.44%) videos had promotional content, and 4 (5.48%) videos had information about support groups, whereas in another study by Szmuda et al., 67 (66.34%) videos mentioned symptoms of stroke, 58 (57.43%) discussed treatment, and 21 (20.79%) discussed patient experiences [13]. It was also found that 32 (43.84%) videos discussed investigations and 34 (46.58%) videos discussed prevention. Hence, from this study, it can be observed that most of the videos were trying to educate viewers about etiology, symptoms, and treatment, while very few videos were educating them about investigations and tests, mortality, rehabilitation, and support groups. Most of the videos educating about treatment did not discuss the advantages, disadvantages, or risks associated with them. They were providing an overview of different treatment modalities and did not go much in depth. However, the good thing is that only a few videos included promotional content. Thus, it can be said that the uploaders were more concerned about trying to educate the viewers instead of promoting pharmaceutical companies or devices, making the videos more educational than a means of promotion.

Our study had several limitations. First, only 105 videos were evaluated, out of which 73 videos were taken into this study, after following inclusion and exclusion criteria. Although there are several videos available on YouTube about stroke, a viewer will watch only the top-performing videos, and thus, we included only the top videos. Second, the total number of likes, comments, and views changes over time. Third, there may be interobserver differences in the GQS and DS for the same video. Same video was not evaluated by more than one author, and duplicates were removed.

## Conclusions

YouTube can become an important source to disseminate information about health-related conditions like stroke if the videos uploaded are of high quality (GQS score) and reliable (DS). The videos uploaded by uploaders other than doctors, hospitals, and healthcare organizations need strict evaluation for quality and reliability before or after they are uploaded, to ensure that correct information is shared with viewers.

## Appendices

**Table 5:** Data collected by authors.

Serial No.	Your search word	Is the post relevant to the topic ?	Title of Video	Posted by	Link to the video	Posted within	Posted by	Duration of video ( example 00:12:29)	Number of views (absolute number without comma or plus)	No of likes (mention absolute number without comma)	Absolute number of comments (absolute number)	Description of SYMPTOMS	Information about cause/etiology ?	Info about INVESTIGATIONS / TESTS	Info about PREVENTION / VACCINES	Info about TREATMENT	Info about MORTALITY	Info about REHABILITATION	Info about SUPPORT GROUPS	Info about PEOPLE/PATIENT'S SHARING THEIR OWN EXPERIENCE	Info about PARENT SHARING THEIR EXPERIENCE WITH THEIR family members	The post has a PROMOTIONAL content by pharmaceutical company or by doctors ?	Global Quality Score	Reliability score (DISCERN ) The answer to each question as "yes" is scored 1 and "no" as 0. Total score is then calculated.
1	StrokeTreatment	Yes	Stroke Diagnosis and Treatment - Acute and Long Term Treatment of Ischemic and Hemorrhagic Stroke	Rhesus Medicine	https://youtu.be/-idw58Yo4Do	More than one year (> 365 days)	Other	00:08:13	78000	1000	27	No	No	Yes	Yes	Yes	No	Yes	No	No	No	No	4	4
2	StrokeTreatment	Yes	0:27 / 2:03 • Stroke Stroke - Causes, Symptoms and Treatment Options	Rehealthify	https://youtu.be/63Wpv2AcaXY	More than one year (> 365 days)	Other	00:02:03	596497	3900	259	yes	Yes	No	No	Yes	No	No	No	No	No	No	2	3
3	StrokeTreatment	Yes	Ischemic Stroke - causes, symptoms, diagnosis, treatment, pathology	Osmosis from Elsevier	https://youtu.be/2IgFri0B85Q	More than one year (> 365 days)	Hospital (Name of hospital on video or description	00:13:39	855907	13000	332	yes	Yes	Yes	Yes	Yes	No	No	No	No	No	No	4	3
4	StrokeTreatment	Yes	Brain Stroke Symptoms And Treatment | Best Treatment for Brain Stroke in Hyderabad, India	Yashoda Hospitals - Hyderabad	https://youtu.be/Ig-MNjHaqvg	More than one year (> 365 days)	Hospital (Name of hospital on video or description	00:02:30	83000	678	44	yes	Yes	No	Yes	Yes	Yes	No	No	No	No	No	4	3
5	StrokeTreatment	Yes	Acute Stroke Treatments	Cleveland Clinic	https://youtu.be/KO3zxqUrNks	More than one year (> 365 days)	Hospital (Name of hospital on video or description	00:02:19	36000	295	23	yes	Yes	Yes	No	Yes	No	No	No	No	No	No	2	3
6	StrokeTreatment	Yes	Emergency Treatment for Ischemic Stroke - Dr. Reza Jahan | UCLA Interventional Radiology	UCLA Health	https://youtu.be/n1qL12UGJt0	More than one year (> 365 days)	Hospital (Name of hospital on video or description	00:14:49	281000	3600	285	yes	Yes	Yes	No	Yes	No	No	No	No	No	Yes	3	3
7	StrokeTreatment	Yes	Brain Stroke, Types of, Causes, Pathology, Symptoms, Treatment and Prevention, Animation.	Alila Medical Media	https://youtu.be/EY98RInP-A4	More than one year (> 365 days)	Other	00:03:46	1736299	18000	599	yes	Yes	No	Yes	Yes	No	No	No	No	No	No	4	3
8	StrokeTreatment	Yes	Brain Stroke Treatment | Best Neurologist In Bangalore - Dr. Sreekanta Swamy | Aster RV Hospital	Aster Hospitals, Bangalore	https://youtu.be/s0KI8sEFIpc	More than one year (> 365 days)	Doctor (video or description mentions doctor's name with "MD" written after it	00:07:06	82000	971	0	No	Yes	Yes	No	Yes	No	No	No	No	No	Yes	2	2
9	StrokeTreatment	Yes	A New Treatment for Stroke Patients When Every Second Counts | NBC Nightly News	NBC News	https://youtu.be/EDr76YzTxHo	More than one year (> 365 days)	News channel (Name of news channel should be in decription)	00:02:21	108000	850	81	yes	No	No	No	Yes	No	No	No	Yes	No	No	2	1
10	StrokeTreatment	Yes	Take This IMMEDIATELY after a Stroke	Dr. Eric Berg DC	https://youtu.be/iBDYdTaMUTg	More than a month to six months (31 - 180 days old)	Doctor (video or description mentions doctor's name with "MD" written after it	00:07:39	1350367	44000	2477	No	No	No	Yes	No	No	No	No	No	No	No	3	2
11	Paralysis	Yes	Paralysis Attack(Stroke)| Dr Ajay Kumar Mishra	Narayana Health	https://www.youtube.com/watch?v=FagWTbulrDA	More than a month to six months (31 - 180 days old)	Hospital (Name of hospital on video or description	00:03:02	22000	268	37	yes	No	Yes	No	Yes	Yes	No	No	No	No	No	3	3
12	Paralysis	Yes	Causes of Paralysis	AllHealthGo	https://www.youtube.com/watch?v=DKjydsx2v8Y	More than one year (> 365 days)	News channel (Name of news channel should be in decription)	00:02:13	7889	83	4	yes	Yes	No	No	No	No	Yes	No	No	No	No	3	4
13	Paralysis	Yes	Paralysis Recovery| Stroke Rehabilitation| Mr.Sachdeva's Story	Nightingales Home Health Services	https://www.youtube.com/watch?v=nVmV7BaYhXM	More than one year (> 365 days)	Other	00:03:41	41397	407	24	yes	No	No	No	Yes	No	Yes	No	Yes	No	No	4	3
14	Paralysis	Yes	Stroke Paralysis Rehabilitation	Detroit Medical Center	https://www.youtube.com/watch?v=_shMPx_r-PM	More than one year (> 365 days)	Hospital (Name of hospital on video or description	00:02:53	262000	0	0	No	No	No	No	Yes	No	Yes	No	Yes	No	Yes	2	3
15	Paralysis	Yes	Exercises For Stroke Related Facial Palsy	Health Q	https://www.youtube.com/watch?v=Px2OgHQMya0	More than one year (> 365 days)	Other	00:07:23	174000	2800	113	yes	Yes	No	No	No	No	Yes	No	No	No	No	3	3
16	Paralysis	Yes	Paralysis:Causes,Signs and Symptoms,Types	Doctor's Circle Worlds Largest Health Platform	https://www.youtube.com/watch?v=9r0ebb-QM6E	More than one year (> 365 days)	Doctor (video or description mentions doctor's name with "MD" written after it	00:05:20	45792	392	52	yes	Yes	No	No	No	No	No	No	No	No	No	5	4
17	Paralysis	Yes	Stroke Hand Paralysis Rehab Treatment India	Advanced Rehab Technologies	https://www.youtube.com/watch?v=T-rl8R0VTWw	More than one year (> 365 days)	Other	00:04:21	3100	4	0	No	No	No	No	Yes	No	Yes	No	No	No	Yes	3	3
18	Paralysis	Yes	Stroke||Paralysis||Brain Attack||Cerebrovascular accident- symptoms and signs	Amit Gauli	https://www.youtube.com/watch?v=BgKMBbdOYxk	More than one year (> 365 days)	Other	00:02:30	3064	10	0	yes	Yes	No	No	Yes	No	No	No	No	No	No	3	3
19	StrokeCure	Yes	A New Treatment for Stroke Patients When Every Second Counts | NBC Nightly News	NBC News	https://youtu.be/EDr76YzTxHo	More than one year (> 365 days)	News channel (Name of news channel should be in decription)	00:02:21	108000	850	81	yes	No	No	No	Yes	No	No	Yes	Yes	No	No	4	4
20	StrokePrevention	Yes	Stroke Prevention	Cleveland Clinic	https://www.youtube.com/watch?v=FqNPUUmzkBI	More than one year (> 365 days)	Hospital (Name of hospital on video or description	00:01:31	1400	15	1	No	Yes	No	Yes	No	No	No	No	No	No	No	4	3
21	StrokePrevention	Yes	Exercise and Stroke Prevention – Barriers to Success	MassGeneralHospital	https://www.youtube.com/watch?v=Pr_eY4rYFtM	More than one year (> 365 days)	Hospital (Name of hospital on video or description	00:03:13	26556	342	15	No	No	No	Yes	No	No	Yes	No	Yes	No	No	4	3
22	StrokePrevention	Yes	8 Steps to Prevent Stroke	Saint Luke's Health System	https://www.youtube.com/watch?v=nymJxw_LULA	More than one year (> 365 days)	Hospital (Name of hospital on video or description	00:01:58	42979	656	31	No	No	No	Yes	No	No	No	No	No	No	No	5	3
23	StrokeCure	Yes	New stroke treatment breakthrough using drug used for treating arthritis | 7NEWS	7NEWS Australia	https://youtu.be/pjqlXl2h_Nk	More than one year (> 365 days)	News channel (Name of news channel should be in decription)	00:05:58	27000	619	196	yes	No	Yes	No	Yes	No	Yes	No	Yes	Yes	No	4	4
24	StrokePrevention	Yes	Stroke: Causes, Risk Factors, Treatment, and Prevention | Mass General Brigham	Mass General Brigham	https://www.youtube.com/watch?v=E_3LSi8QOKA	More than one year (> 365 days)	Hospital (Name of hospital on video or description	00:11:24	15634	223	0	yes	Yes	Yes	Yes	Yes	Yes	No	No	No	No	No	5	4
25	StrokePrevention	Yes	What to Eat for Stroke Prevention	NutritionFacts.org	https://www.youtube.com/watch?v=G1E7Upc2lPQ	More than one year (> 365 days)	Doctor (video or description mentions doctor's name with "MD" written after it	00:04:51	80822	2800	189	No	No	No	Yes	No	Yes	No	No	No	No	No	4	4
26	StrokeCure	Yes	Turmeric: A Hope for Stroke Patients	The University of Arizona	https://youtu.be/reQM1epROXk	More than one year (> 365 days)	Other	00:03:54	344872	49000	366	No	No	Yes	Yes	Yes	No	Yes	No	No	No	No	3	3
27	StrokePrevention	Yes	0:18 / 1:19 Four things you can do to prevent stroke	Hindustan Times	https://www.youtube.com/watch?v=DVvKk8MpNJI	More than one year (> 365 days)	News channel (Name of news channel should be in decription)	00:01:19	15794	156	2	No	No	No	Yes	No	Yes	No	No	No	No	No	3	2
28	StrokeCure	Yes	New hope for the treatment of stroke victims	QldBrainInstitute	https://youtu.be/fMJHI4tIWNs	More than one year (> 365 days)	Other	00:06:38	14000	6	0	No	No	Yes	Yes	Yes	No	Yes	No	No	No	Yes	5	4
29	Paralysis	Yes	Bells Palsy and Stroke	The Noted Anatomist	https://youtu.be/qgO8Vhij758	More than one year (> 365 days)	Other	00:15:32	112018	3700	270	yes	Yes	No	No	No	No	No	No	No	No	No	4	3
30	Stroke	Yes	Stroke | Nucleus Health	Nucleus Medical Media	https://youtu.be/pcmrgwNCPwM	More than one year (> 365 days)	Healthcare organization (Like UNICEF, WHO, CDC etc)	00:04:13	6226196	63000	1700	yes	Yes	Yes	No	Yes	No	No	No	No	No	No	4	4
31	Stroke	Yes	Mayo Clinic Explains Strokes	Mayo Clinic	https://youtu.be/Ffk-L9ODEO8	More than six months to last one year (180 - 365 days)	Hospital (Name of hospital on video or description	00:05:35	113321	270	0	yes	Yes	Yes	Yes	Yes	Yes	No	No	No	No	No	5	5
32	Stroke	Yes	What is a Stroke?	The Photoprotection Channel	https://youtu.be/BKE8OYqzacQ	More than one year (> 365 days)	Other	00:12:12	4107	52	2	yes	Yes	Yes	No	Yes	No	No	No	No	No	No	4	4
33	Stroke	Yes	What is a Stroke? (HealthSketch)	HealthSketch	https://youtu.be/ryIGnzodxDs	More than one year (> 365 days)	Other	00:05:40	1583867	21000	802	yes	Yes	Yes	Yes	Yes	Yes	No	Yes	No	No	No	5	4
34	Stroke	Yes	Stroke Survivor, 28, Shares Symptoms And Warning Signs	Today	https://youtu.be/ZjsAR7eo4T4	More than six months to last one year (180 - 365 days)	News channel (Name of news channel should be in decription)	00:05:05	37633	387	41	yes	No	No	No	No	No	No	No	Yes	No	No	4	4
35	Stroke	Yes	Ischemic Stroke | Lisa Klein	Johns Hopkins Medicine	https://youtu.be/m4asiIqG6dY	More than one year (> 365 days)	Hospital (Name of hospital on video or description	00:11:29	12244	153	9	yes	Yes	Yes	Yes	Yes	Yes	No	No	No	No	No	5	4
36	Stroke	Yes	Hemorrhagic Stroke | Lisa Klein	Johns Hopkins Medicine	https://youtu.be/f8HSNJr3sVc	More than one year (> 365 days)	Hospital (Name of hospital on video or description	00:10:32	23274	273	14	yes	Yes	Yes	Yes	Yes	Yes	No	No	No	No	No	5	4
37	Stroke	Yes	Stroke: Acute Stroke Intervention	Norton Healthcare	https://youtu.be/Cr9Pb3liSlA	More than one year (> 365 days)	Healthcare organization (Like UNICEF, WHO, CDC etc)	00:02:39	370499	2200	63	No	Yes	No	No	Yes	No	No	No	No	No	No	3	4
38	Stroke	Yes	Stroke & Cerebrovascular Accident (CVA) - Medical-Surgical - Nervous System | @LevelUpRN	Level Up RN	https://youtu.be/xPDtaEXHuiM	More than one year (> 365 days)	Other	00:06:28	36241	888	31	yes	Yes	Yes	Yes	Yes	No	No	No	No	No	No	4	4
39	Stroke support	Yes	Stroke - causes, symptoms and treatment	Realhealthify	https://youtu.be/63Wpv2AcaXY	More than one year (> 365 days)	Other	00:02:04	596000	3900	259	yes	No	No	No	Yes	No	No	No	No	No	No	4	4
40	Stroke	Yes	6 Warning Signs of a Stroke | Cleveland Clinic	Cleveland Clinic	https://youtu.be/2Z_zKxSqgCo	More than six months to last one year (180 - 365 days)	Hospital (Name of hospital on video or description	00:02:37	74013	481	17	yes	No	No	No	No	Yes	No	No	No	No	No	3	4
41	Stroke support	Yes	Signs of Stroke | Think F.A.S.T.E.R.	Beaumont Health	https://youtu.be/wkXJOUe5G60	More than one year (> 365 days)	Hospital (Name of hospital on video or description	00:08:12	85000	630	0	yes	Yes	No	Yes	Yes	Yes	No	Yes	Yes	Yes	No	5	5
42	Stroke support	Yes	Reduce Your Risk of Stroke	Mayfield Brain & Spine	https://youtu.be/sdcqchFK74o	More than one year (> 365 days)	Hospital (Name of hospital on video or description	00:05:38	97000	0	2	yes	Yes	Yes	Yes	Yes	Yes	No	No	No	No	No	5	5
43	Stroke support	Yes	How to Prevent TIA (Transient Ischaemic Attack) Mini-stroke?	Dr. Eric Berg DC	https://youtu.be/NEQsqwT3xYk	More than one year (> 365 days)	Doctor (video or description mentions doctor's name with "MD" written after it	00:06:07	308000	10000	570	yes	Yes	No	Yes	Yes	No	No	No	No	No	No	5	5
44	Stroke support	Yes	What is stroke?	FreeMedEducation	https://youtu.be/NAPgHluIuNE	More than one year (> 365 days)	Other	00:03:17	20000	488	9	yes	Yes	No	Yes	Yes	Yes	Yes	No	No	No	No	5	5
45	Stroke support	Yes	Stroke Symptoms - Mayo Clinic	Mayo Clinic	https://youtu.be/UPJQXyBQr88	More than one year (> 365 days)	Hospital (Name of hospital on video or description	00:02:52	33000	181	0	yes	No	No	No	No	No	No	No	No	No	No	3	4
46	Stroke support	Yes	Biggest Risk Factor for Stroke	Doctor Mike Hansen	https://youtu.be/xbEpjWGH9z4	More than a month to six months (31 - 180 days old)	Doctor (video or description mentions doctor's name with "MD" written after it	00:06:15	120000	3900	653	yes	Yes	No	Yes	No	No	No	No	No	Yes	No	4	4
47	Stroke support	Yes	Are you at Risk for a Stroke? Learn the warning signs!	National Stroke Association	https://youtu.be/CttD9H_G9Sg	More than one year (> 365 days)	Healthcare organization (Like UNICEF, WHO, CDC etc)	00:02:12	52000	195	14	yes	Yes	No	Yes	No	Yes	No	No	No	No	No	4	4
48	Stroke support	Yes	Stroke signs	Pfizer	https://m.youtube.com/watch?v=mBPJp7H4bo8	More than one year (> 365 days)	Other	00:05:04	529000	3400	0	yes	Yes	No	Yes	No	Yes	No	No	No	No	No	5	5
49	Stroke support	Yes	Stroke - Not Just An Old Person's Disease	Pfizer	https://m.youtube.com/watch?v=TaxAMJQIPFY	More than one year (> 365 days)	Other	00:05:06	55000	351	0	yes	Yes	No	Yes	Yes	Yes	Yes	No	Yes	No	No	5	5
50	StrokeCure	Yes	Reverse Stroke | 60 Minutes	60 minutes Australia	https://youtu.be/-nEtQ-Wxkas	More than one year (> 365 days)	News channel (Name of news channel should be in decription)	00:22:03	1000000	26000	0	yes	No	Yes	No	Yes	No	Yes	No	Yes	Yes	Yes	5	5
51	StrokeCure	Yes	Meet a pioneer in stroke recovery	CBS sunday morning	https://youtu.be/GNJTrtNxTFk	More than one year (> 365 days)	News channel (Name of news channel should be in decription)	00:07:13	47000	904	75	yes	No	Yes	No	Yes	No	Yes	No	Yes	Yes	Yes	4	5
52	StrokeCure	Yes	A Life Changed: A new treatment for stroke	Institute of Neurological Recovery	https://youtu.be/jXHSsMQsq1k	More than one year (> 365 days)	Hospital (Name of hospital on video or description	00:17:28	144000	0	72	yes	No	Yes	No	Yes	No	Yes	No	Yes	Yes	Yes	3	4
53	StrokeCure	Yes	Stem cell therapy to treat acute stroke patients	VJNeurology	https://youtu.be/cRZO9-kFYj4	More than six months to last one year (180 - 365 days)	Other	00:02:11	3225	51	0	No	No	Yes	No	Yes	No	No	No	No	No	Yes	2	2
54	StrokeCure	Yes	Stem cells used to restore mobility in stroke patients	NJ Spotlight News	https://youtu.be/kwDqYSWVueo	More than one year (> 365 days)	News channel (Name of news channel should be in decription)	00:03:04	74000	114	17	yes	Yes	Yes	No	Yes	No	Yes	Yes	Yes	Yes	Yes	5	5
55	StrokePrevention	Yes	WAYS TO PREVENT STROKE YOU MIGHT NOT KNOW	Ivanhoe Web	https://www.youtube.com/watch?v=Tk2u9SmvxLM	More than one year (> 365 days)	Other	00:01:18	2721	34	0	No	Yes	No	Yes	No	No	No	No	No	No	No	3	3
56	StrokePrevention	Yes	How to Prevent A Stroke with Dr. Richard Green	Columbia University Department of Surgery	https://www.youtube.com/watch?v=nRVqtAvDjWk	More than one year (> 365 days)	Hospital (Name of hospital on video or description	00:02:41	30066	379a	27	No	Yes	No	Yes	No	Yes	No	No	No	No	No	4	3
57	StrokePrevention	Yes	Reduce Your Stroke Risk: ABCs of Stroke Prevention	El Camino Health	https://www.youtube.com/watch?v=REKZ5paebXM	More than one year (> 365 days)	Hospital (Name of hospital on video or description	00:04:31	41329	564	0	No	Yes	No	Yes	No	No	No	No	No	No	No	5	3
58	StrokePrevention	Yes	Reduce Your Risk of Stroke	Mayfield Brain & Spine	https://www.youtube.com/watch?v=sdcqchFK74o	More than one year (> 365 days)	Hospital (Name of hospital on video or description	00:05:37	97683	1	2	No	Yes	No	Yes	No	No	No	No	No	No	No	5	3
59	Stroke	Yes	Ischemic and hemorrhagic stroke	Ninja nerd	https://youtu.be/7lpqxDEfszY	More than one year (> 365 days)	Doctor (video or description mentions doctor's name with "MD" written after it	00:38:59	176695	6900	176	yes	Yes	Yes	No	Yes	No	No	No	No	No	No	4	4
60	StrokeCause	Yes	What causes ischemic stroke and how to treat	Prism health	https://youtu.be/1k_PCpsvrtc	More than one year (> 365 days)	Doctor (video or description mentions doctor's name with "MD" written after it	5:04	7270	88	1	yes	Yes	No	No	Yes	No	No	No	No	No	No	4	3
61	StrokeCause	Yes	Duke neurologist diagnoses tricky underlying cause of stroke in a young patient	Duke Health	https://youtu.be/_mStDz3kgts	More than one year (> 365 days)	Hospital (Name of hospital on video or description	3:43	7861	97	0	yes	Yes	Yes	No	Yes	No	No	No	Yes	Yes	No	4	5
62	StrokeCause	Yes	Did you have a stroke? - F.A.S.T	How to meditate	https://youtu.be/b70Plv_n9iA	More than six months to last one year (180 - 365 days)	Doctor (video or description mentions doctor's name with "MD" written after it	00:8:50	1534	60	5	yes	Yes	Yes	Yes	Yes	No	No	No	No	No	No	5	5
63	StrokeCause	Yes	Biggest risk factor for stroke	Doctor Mike hansen	https://youtu.be/xbEpjWGH9z4	More than a month to six months (31 - 180 days old)	Doctor (video or description mentions doctor's name with "MD" written after it	00:6:15	120113	3900	653	yes	Yes	No	Yes	No	No	No	No	No	No	No	5	3
64	StrokeCause	Yes	The early show -stroke prevention: An apple a day	CBS News	https://youtu.be/chMQS4Zr_ps	More than one year (> 365 days)	News channel (Name of news channel should be in decription)	00:02:51	4820000	326	11	No	Yes	No	Yes	No	No	No	No	No	No	No	3	2
65	StrokeCure	Yes	Understanding stroke symptoms and causes|Dr.Veena	Narayana Health	https://youtu.be/uMUP1U0lKsY	More than one year (> 365 days)	Doctor (video or description mentions doctor's name with "MD" written after it	00:03:18	695000	48	3	yes	Yes	Yes	Yes	Yes	No	No	No	No	No	No	4	5
66	StrokeCause	Yes	What causes a brain stroke? Brain attack | The Dr Binocs Show | Peekaboo Kidz	Peekaboo Kidz	https://youtu.be/yrcnARiD4Ag	More than one year (> 365 days)	Doctor (video or description mentions doctor's name with "MD" written after it	00:05:37	3470000	7700	0	yes	Yes	No	No	Yes	No	No	No	No	No	No	3	3
67	Paralysis	Yes	Jame's Story: Recovery from Left Side Paralysis After Stroke	Flint Rehab	https://youtu.be/hW0pDCPsVc4	More than one year (> 365 days)	Other	00:01:40	51139	521	118	yes	Yes	No	No	No	No	Yes	No	Yes	No	Yes	2	3
68	Paralysis	Yes	Teen Girl Paralysed After Stroke Learns To Walk Again	Loma Linda University Health	https://youtu.be/Jd-3vDcjUJg	More than six months to last one year (180 - 365 days)	Hospital (Name of hospital on video or description	00:12:24	15000	205	8	yes	Yes	Yes	No	Yes	Yes	Yes	No	Yes	Yes	No	4	5
69	StrokeCure	Yes	What causes ischemic stroke and how to treat	Prisma Health	https://youtu.be/1k_PCpsvrtc	More than one year (> 365 days)	Hospital (Name of hospital on video or description	00:05:04	74000	90	1	yes	Yes	Yes	No	Yes	No	Yes	No	No	No	Yes	4	4
70	Stroke support	Yes	Emergency Medical Stroke Assessment (EMSA)	UAB Medicine	https://youtu.be/ZNenSdNaM-E	More than one year (> 365 days)	Doctor (video or description mentions doctor's name with "MD" written after it	00:07:43	17000	333	10	yes	No	Yes	No	Yes	Yes	No	No	No	No	No	4	4
71	StrokeCause	Yes	Hemorrhagic stroke: causes and treatments	Prisma health	https://youtu.be/wAwvAsR1UTo	More than one year (> 365 days)	Doctor (video or description mentions doctor's name with "MD" written after it	00:04:17	4830	236	7	yes	Yes	Yes	No	Yes	No	No	No	No	No	No	4	4
72	StrokeCause	Yes	What happens during a stroke?- Vibhav Goswami	TED-Ed	https://youtu.be/-NJm4TJ2it0	More than one year (> 365 days)	Doctor (video or description mentions doctor's name with "MD" written after it	00:04:59	1,85,00,000	750000	21000	yes	Yes	No	No	Yes	No	No	No	No	No	No	4	3
73	StrokeCause	Yes	Hemorrhagic stroke: intracerebral hemorrhage- causes, symptoms, diagnosis, treatment, pathology	Osmosis from Elsevier	https://youtu.be/1BBul_LC1cE	More than six months to last one year (180 - 365 days)	Hospital (Name of hospital on video or description	00:09:40	27,70,000	1400	47	yes	Yes	Yes	No	Yes	Yes	No	No	No	No	No	4	5
